# Dichlorido{*N*,*N*-dimethyl-*N*′-[1-(2-pyrid­yl)ethyl­idene]ethane-1,2-diamine-κ^3^ *N*,*N*′,*N*′′}manganese(II)

**DOI:** 10.1107/S1600536811002030

**Published:** 2011-01-22

**Authors:** Nurul Azimah Ikmal Hisham, Nura Suleiman Gwaram, Hamid Khaledi, Hapipah Mohd Ali

**Affiliations:** a Department of Chemistry, University of Malaya, 50603 Kuala Lumpur, Malaysia

## Abstract

The asymmetric unit of the title compound, [MnCl_2_(C_11_H_17_N_3_)], contains two crystallographically independent mol­ecules with slightly different geometries. In each mol­ecule, the Mn^II^ ion is five coordinated by the *N*,*N*′,*N*′′-tridentate Schiff base and two Cl atoms in a distorted square-pyramidal geometry. In the crystal, C—H⋯Cl hydrogen bonds link adjacent mol­ecules into a three-dimensional network.

## Related literature

For the structure of a CuCl_2_ complex of the same Schiff base, see: Saleh Salga *et al.* (2010) ▶. For structures of similar Mn^II^ complexes, see: Gibson *et al.* (2003 ▶); Reardon *et al.* (2002 ▶).
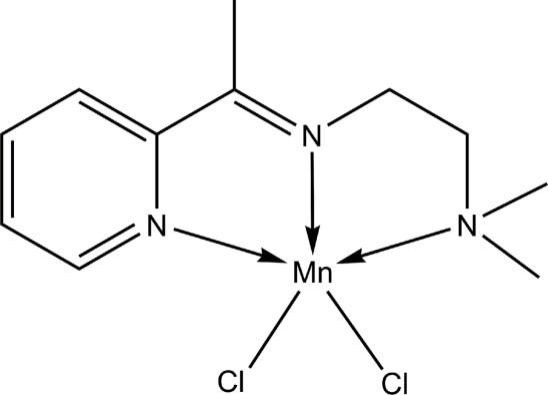


## Experimental

### 

#### Crystal data

[MnCl_2_(C_11_H_17_N_3_)]
*M* *_r_* = 317.12
Monoclinic, 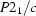

*a* = 17.6157 (8) Å
*b* = 9.9269 (4) Å
*c* = 20.4710 (8) Å
β = 124.592 (3)°
*V* = 2946.9 (2) Å^3^
*Z* = 8
Mo *K*α radiation
μ = 1.24 mm^−1^
*T* = 100 K
0.19 × 0.13 × 0.09 mm

#### Data collection

Bruker APEXII CCD diffractometer
Absorption correction: multi-scan (*SADABS*; Sheldrick, 1996 ▶) *T*_min_ = 0.798, *T*_max_ = 0.897
26611 measured reflections
6426 independent reflections
5326 reflections with *I* > 2σ(*I*)
*R*_int_ = 0.043

#### Refinement

*R*[*F* ^2^ > 2σ(*F* ^2^)] = 0.027
*wR*(*F* ^2^) = 0.063
*S* = 1.02
6426 reflections
313 parameters
H-atom parameters constrained
Δρ_max_ = 0.32 e Å^−3^
Δρ_min_ = −0.32 e Å^−3^

### 

Data collection: *APEX2* (Bruker, 2007 ▶); cell refinement: *SAINT* (Bruker, 2007 ▶); data reduction: *SAINT*; program(s) used to solve structure: *SHELXS97* (Sheldrick, 2008 ▶); program(s) used to refine structure: *SHELXL97* (Sheldrick, 2008 ▶); molecular graphics: *X-SEED* (Barbour, 2001 ▶); software used to prepare material for publication: *SHELXL97* and *publCIF* (Westrip, 2010 ▶).

## Supplementary Material

Crystal structure: contains datablocks I, global. DOI: 10.1107/S1600536811002030/is2666sup1.cif

Structure factors: contains datablocks I. DOI: 10.1107/S1600536811002030/is2666Isup2.hkl

Additional supplementary materials:  crystallographic information; 3D view; checkCIF report

## Figures and Tables

**Table 1:** Hydrogen-bond geometry (Å, °)

*D*—H⋯*A*	*D*—H	H⋯*A*	*D*⋯*A*	*D*—H⋯*A*
C4—H4⋯Cl4^i^	0.95	2.73	3.6115 (18)	155
C7—H7*B*⋯Cl3^i^	0.98	2.75	3.7280 (18)	175
C14—H14⋯Cl4^ii^	0.95	2.82	3.7048 (18)	156
C19—H19*A*⋯Cl3^iii^	0.99	2.64	3.5839 (18)	159
C19—H19*B*⋯Cl4^i^	0.99	2.73	3.6579 (19)	156
C22—H22*B*⋯Cl4^i^	0.98	2.78	3.6693 (19)	151

Symmetry codes: (i) 
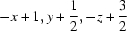
; (ii) 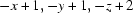; (iii) 
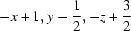
.

**Fig. 1.**
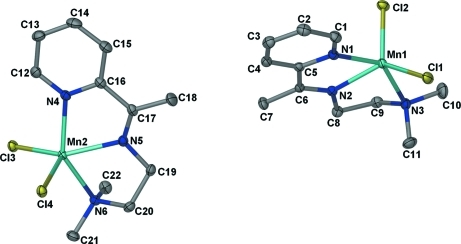
Displacement ellipsoid plot of the title compound at the 50% probability level. Hydrogen atoms have been omitted for clarity.

## supplementary crystallographic information

### Comment

The title compound was obtained upon complexation of the Schiff base, *N,N*-dimethyl-*N'*-[methyl(2-pyridyl)methylene]ethane-1,2-diamine, with MnCl_2_. Similar to its analogous copper(II) complex (Saleh Salga *et al.*, 2010), the metal center is five-coordinated by the *N,N',N*"-tridentate Schiff base and two Cl atoms. Two geometrically different molecules exist in the crystal structure. In both molecules, the Mn^II^ ions are in a square-pyramidal coordination environment with different degrees of distortion from the ideal geometry as revealed by the τ values of 0.101 for Mn1 complex and 0.035 for Mn2 complex. The weighted r.m.s. fit for the superposition of the non-H atoms in both molecules is 0.0868 Å. The Mn—Cl and Mn—N bond lengths in the two molecules are comparable with those in the related structures (Gibson *et al.*, 2003; Reardon *et al.*, 2002). In the crystal, the adjacent molecules are connected *via* C—H···Cl hydrogen bonds into a three-dimensional polymeric structure. The crystal structure contains void spaces with the size of 54.00 Å^-3^ within which there is no evidence for included solvent.

### Experimental

A mixture of 2-acetylpyridine (0.61 g, 5 mmol) and *N,N*-dimethylethyldiamine (0.44 g, 5 mmol) in ethanol (50 ml) was refluxed for 2 hr followed by addition of a solution of manganese(II) chloride (0.63 g, 5 mmol) in a minimum amount of water. The resulting solution was refluxed for 30 min, then set aside at room temperature. The crystals of the title compound were obtained after a few days.

### Refinement

Hydrogen atoms were placed at calculated positions (C—H 0.95–0.99 Å) and were treated as riding on their parent atoms, with *U*_iso_(H) set to 1.2–1.5 times *U*_eq_(C).

### Crystal data

**Table:**

[MnCl_2_(C_11_H_17_N_3_)]	*F*(000) = 1304
*M**_r_* = 317.12	*D*_x_ = 1.430 Mg m^−^^3^
Monoclinic, *P*2_1_/*c*	Mo *K*α radiation, λ = 0.71073 Å
Hall symbol: -P 2ybc	Cell parameters from 7274 reflections
*a* = 17.6157 (8) Å	θ = 2.4–29.6°
*b* = 9.9269 (4) Å	µ = 1.24 mm^−^^1^
*c* = 20.4710 (8) Å	*T* = 100 K
β = 124.592 (3)°	Block, brown
*V* = 2946.9 (2) Å^3^	0.19 × 0.13 × 0.09 mm
*Z* = 8	

### Data collection

**Table:**

Bruker APEXII CCD diffractometer	6426 independent reflections
Radiation source: fine-focus sealed tube	5326 reflections with *I* > 2σ(*I*)
graphite	*R*_int_ = 0.043
φ and ω scans	θ_max_ = 27.0°, θ_min_ = 2.0°
Absorption correction: multi-scan (*SADABS*; Sheldrick, 1996)	*h* = −22→22
*T*_min_ = 0.798, *T*_max_ = 0.897	*k* = −12→12
26611 measured reflections	*l* = −26→24

### Refinement

**Table:**

Refinement on *F*^2^	Primary atom site location: structure-invariant direct methods
Least-squares matrix: full	Secondary atom site location: difference Fourier map
*R*[*F*^2^ > 2σ(*F*^2^)] = 0.027	Hydrogen site location: inferred from neighbouring sites
*wR*(*F*^2^) = 0.063	H-atom parameters constrained
*S* = 1.02	*w* = 1/[σ^2^(*F*_o_^2^) + (0.0284*P*)^2^ + 0.699*P*] where *P* = (*F*_o_^2^ + 2*F*_c_^2^)/3
6426 reflections	(Δ/σ)_max_ = 0.004
313 parameters	Δρ_max_ = 0.32 e Å^−^^3^
0 restraints	Δρ_min_ = −0.32 e Å^−^^3^

### Special details

**Table:**

Geometry. All e.s.d.'s (except the e.s.d. in the dihedral angle between two l.s. planes) are estimated using the full covariance matrix. The cell e.s.d.'s are taken into account individually in the estimation of e.s.d.'s in distances, angles and torsion angles; correlations between e.s.d.'s in cell parameters are only used when they are defined by crystal symmetry. An approximate (isotropic) treatment of cell e.s.d.'s is used for estimating e.s.d.'s involving l.s. planes.
Refinement. Refinement of *F*^2^ against ALL reflections. The weighted *R*-factor *wR* and goodness of fit *S* are based on *F*^2^, conventional *R*-factors *R* are based on *F*, with *F* set to zero for negative *F*^2^. The threshold expression of *F*^2^ > σ(*F*^2^) is used only for calculating *R*-factors(gt) *etc*. and is not relevant to the choice of reflections for refinement. *R*-factors based on *F*^2^ are statistically about twice as large as those based on *F*, and *R*- factors based on ALL data will be even larger.

### Fractional atomic coordinates and isotropic or equivalent isotropic displacement parameters (Å^2^)

**Table:**

	*x*	*y*	*z*	*U*_iso_*/*U*_eq_	
Mn1	1.124031 (16)	0.97384 (3)	0.858429 (14)	0.01582 (7)	
Cl1	1.23612 (3)	0.80041 (5)	0.91747 (2)	0.02316 (10)	
Cl2	1.15773 (3)	1.20051 (5)	0.90329 (2)	0.02196 (10)	
N1	1.04215 (9)	0.90231 (15)	0.90492 (8)	0.0168 (3)	
N2	0.97678 (9)	0.99030 (14)	0.76157 (8)	0.0160 (3)	
N3	1.12012 (10)	0.99132 (15)	0.74495 (9)	0.0197 (3)	
C1	1.07802 (12)	0.85447 (19)	0.97795 (10)	0.0216 (4)	
H1	1.1430	0.8435	1.0125	0.026*	
C2	1.02463 (13)	0.8203 (2)	1.00538 (10)	0.0242 (4)	
H2	1.0526	0.7877	1.0579	0.029*	
C3	0.93044 (12)	0.83419 (19)	0.95525 (10)	0.0234 (4)	
H3	0.8922	0.8111	0.9726	0.028*	
C4	0.89186 (12)	0.88283 (19)	0.87851 (10)	0.0200 (4)	
H4	0.8270	0.8929	0.8427	0.024*	
C5	0.94953 (11)	0.91610 (18)	0.85543 (9)	0.0156 (3)	
C6	0.91493 (11)	0.96556 (17)	0.77415 (9)	0.0153 (3)	
C7	0.81348 (11)	0.98027 (19)	0.71441 (10)	0.0213 (4)	
H7A	0.8021	1.0261	0.6672	0.032*	
H7B	0.7871	1.0334	0.7373	0.032*	
H7C	0.7847	0.8910	0.6995	0.032*	
C8	0.95400 (11)	1.03545 (19)	0.68423 (10)	0.0188 (4)	
H8A	0.9082	1.1093	0.6636	0.023*	
H8B	0.9274	0.9602	0.6457	0.023*	
C9	1.04255 (12)	1.08444 (19)	0.69561 (10)	0.0199 (4)	
H9A	1.0325	1.0940	0.6431	0.024*	
H9B	1.0585	1.1743	0.7211	0.024*	
C10	1.20578 (13)	1.0510 (2)	0.76109 (12)	0.0322 (5)	
H10A	1.1994	1.0662	0.7109	0.048*	
H10B	1.2574	0.9895	0.7944	0.048*	
H10C	1.2175	1.1371	0.7888	0.048*	
C11	1.10379 (13)	0.8605 (2)	0.70528 (11)	0.0289 (4)	
H11A	1.0482	0.8191	0.6965	0.043*	
H11B	1.1568	0.8013	0.7387	0.043*	
H11C	1.0955	0.8740	0.6542	0.043*	
Mn2	0.399031 (16)	0.52217 (3)	0.750325 (14)	0.01472 (7)	
Cl3	0.27334 (3)	0.67387 (4)	0.68459 (2)	0.01975 (9)	
Cl4	0.35006 (3)	0.30314 (4)	0.75588 (2)	0.01934 (9)	
N4	0.46738 (9)	0.59908 (15)	0.87463 (8)	0.0169 (3)	
N5	0.55015 (9)	0.51120 (14)	0.80993 (8)	0.0169 (3)	
N6	0.41710 (9)	0.49145 (14)	0.65054 (8)	0.0168 (3)	
C12	0.42304 (12)	0.65017 (19)	0.90427 (10)	0.0207 (4)	
H12	0.3578	0.6583	0.8703	0.025*	
C13	0.46790 (13)	0.6921 (2)	0.98259 (10)	0.0257 (4)	
H13	0.4340	0.7269	1.0020	0.031*	
C14	0.56266 (13)	0.6821 (2)	1.03155 (10)	0.0272 (4)	
H14	0.5952	0.7099	1.0854	0.033*	
C15	0.60977 (12)	0.6307 (2)	1.00105 (10)	0.0238 (4)	
H15	0.6751	0.6240	1.0337	0.029*	
C16	0.56054 (11)	0.58944 (18)	0.92261 (10)	0.0178 (4)	
C17	0.60500 (11)	0.53567 (18)	0.88397 (10)	0.0182 (4)	
C18	0.70730 (12)	0.5158 (2)	0.93333 (11)	0.0286 (4)	
H18A	0.7239	0.4444	0.9723	0.043*	
H18B	0.7378	0.5999	0.9610	0.043*	
H18C	0.7272	0.4901	0.8990	0.043*	
C19	0.58162 (11)	0.46412 (18)	0.76133 (10)	0.0190 (4)	
H19A	0.6300	0.3947	0.7904	0.023*	
H19B	0.6079	0.5401	0.7490	0.023*	
C20	0.49955 (11)	0.40536 (19)	0.68527 (10)	0.0198 (4)	
H20A	0.5155	0.3942	0.6464	0.024*	
H20B	0.4855	0.3151	0.6964	0.024*	
C21	0.33693 (12)	0.4222 (2)	0.58233 (10)	0.0240 (4)	
H21A	0.2820	0.4788	0.5603	0.036*	
H21B	0.3270	0.3361	0.5998	0.036*	
H21C	0.3485	0.4059	0.5415	0.036*	
C22	0.43085 (12)	0.62049 (19)	0.62313 (10)	0.0235 (4)	
H22A	0.4458	0.6033	0.5845	0.035*	
H22B	0.4817	0.6699	0.6685	0.035*	
H22C	0.3743	0.6742	0.5982	0.035*	

### Atomic displacement parameters (Å^2^)

**Table:**

	*U*^11^	*U*^22^	*U*^33^	*U*^12^	*U*^13^	*U*^23^
Mn1	0.01282 (12)	0.01482 (15)	0.01788 (13)	−0.00021 (10)	0.00756 (10)	−0.00039 (10)
Cl1	0.0172 (2)	0.0201 (3)	0.0250 (2)	0.00366 (17)	0.00763 (17)	−0.00099 (18)
Cl2	0.0208 (2)	0.0159 (2)	0.0226 (2)	−0.00041 (17)	0.00844 (17)	−0.00178 (17)
N1	0.0158 (7)	0.0151 (9)	0.0162 (7)	0.0004 (6)	0.0071 (6)	0.0001 (6)
N2	0.0165 (7)	0.0143 (8)	0.0165 (7)	−0.0002 (6)	0.0090 (6)	0.0007 (6)
N3	0.0177 (7)	0.0193 (9)	0.0244 (8)	−0.0016 (6)	0.0133 (6)	−0.0015 (6)
C1	0.0190 (8)	0.0213 (11)	0.0185 (8)	0.0008 (7)	0.0071 (7)	0.0017 (7)
C2	0.0292 (9)	0.0224 (11)	0.0187 (9)	−0.0008 (8)	0.0122 (8)	0.0030 (8)
C3	0.0296 (9)	0.0216 (11)	0.0263 (9)	−0.0020 (8)	0.0202 (8)	0.0005 (8)
C4	0.0187 (8)	0.0192 (11)	0.0217 (9)	−0.0001 (7)	0.0113 (7)	−0.0018 (7)
C5	0.0166 (8)	0.0115 (9)	0.0170 (8)	0.0003 (6)	0.0085 (7)	−0.0011 (7)
C6	0.0151 (8)	0.0116 (9)	0.0185 (8)	0.0006 (6)	0.0092 (7)	−0.0011 (7)
C7	0.0152 (8)	0.0259 (11)	0.0199 (9)	0.0009 (7)	0.0083 (7)	0.0019 (7)
C8	0.0194 (8)	0.0202 (11)	0.0165 (8)	0.0016 (7)	0.0099 (7)	0.0023 (7)
C9	0.0253 (9)	0.0161 (10)	0.0215 (9)	−0.0013 (7)	0.0152 (7)	0.0013 (7)
C10	0.0257 (10)	0.0407 (14)	0.0371 (11)	−0.0042 (9)	0.0221 (9)	0.0018 (9)
C11	0.0342 (11)	0.0244 (12)	0.0302 (10)	0.0037 (8)	0.0195 (9)	−0.0055 (8)
Mn2	0.01270 (12)	0.01534 (15)	0.01475 (13)	−0.00070 (10)	0.00696 (10)	−0.00102 (10)
Cl3	0.01567 (18)	0.0178 (2)	0.0222 (2)	0.00130 (16)	0.00860 (16)	0.00015 (17)
Cl4	0.01727 (19)	0.0164 (2)	0.0241 (2)	0.00004 (16)	0.01153 (17)	0.00154 (17)
N4	0.0164 (7)	0.0160 (9)	0.0176 (7)	−0.0028 (6)	0.0092 (6)	−0.0010 (6)
N5	0.0157 (7)	0.0154 (9)	0.0195 (7)	0.0004 (6)	0.0099 (6)	0.0007 (6)
N6	0.0171 (7)	0.0153 (9)	0.0166 (7)	−0.0017 (6)	0.0087 (6)	−0.0009 (6)
C12	0.0203 (8)	0.0191 (11)	0.0250 (9)	−0.0040 (7)	0.0143 (7)	−0.0020 (7)
C13	0.0324 (10)	0.0259 (12)	0.0254 (9)	−0.0054 (8)	0.0204 (8)	−0.0064 (8)
C14	0.0340 (10)	0.0276 (12)	0.0173 (9)	−0.0093 (8)	0.0129 (8)	−0.0057 (8)
C15	0.0210 (9)	0.0251 (12)	0.0182 (9)	−0.0038 (8)	0.0069 (7)	−0.0002 (7)
C16	0.0180 (8)	0.0148 (10)	0.0176 (8)	−0.0013 (7)	0.0083 (7)	0.0026 (7)
C17	0.0159 (8)	0.0141 (10)	0.0209 (8)	−0.0006 (7)	0.0082 (7)	0.0023 (7)
C18	0.0160 (9)	0.0335 (13)	0.0261 (10)	0.0028 (8)	0.0059 (8)	−0.0031 (8)
C19	0.0176 (8)	0.0182 (10)	0.0236 (9)	0.0024 (7)	0.0131 (7)	0.0008 (7)
C20	0.0242 (9)	0.0167 (10)	0.0233 (9)	0.0013 (7)	0.0163 (8)	−0.0011 (7)
C21	0.0228 (9)	0.0267 (12)	0.0187 (9)	−0.0051 (8)	0.0095 (7)	−0.0049 (8)
C22	0.0246 (9)	0.0217 (11)	0.0238 (9)	−0.0004 (8)	0.0135 (8)	0.0043 (8)

### Geometric parameters (Å, °)

**Table:**

Mn1—N2	2.2015 (14)	Mn2—N5	2.2134 (13)
Mn1—N1	2.2470 (14)	Mn2—N4	2.2426 (13)
Mn1—N3	2.2911 (14)	Mn2—N6	2.2596 (14)
Mn1—Cl1	2.3694 (5)	Mn2—Cl4	2.3656 (5)
Mn1—Cl2	2.3748 (5)	Mn2—Cl3	2.3659 (5)
N1—C1	1.337 (2)	N4—C12	1.332 (2)
N1—C5	1.353 (2)	N4—C16	1.355 (2)
N2—C6	1.278 (2)	N5—C17	1.275 (2)
N2—C8	1.465 (2)	N5—C19	1.464 (2)
N3—C11	1.471 (2)	N6—C22	1.473 (2)
N3—C10	1.474 (2)	N6—C20	1.474 (2)
N3—C9	1.475 (2)	N6—C21	1.475 (2)
C1—C2	1.384 (2)	C12—C13	1.389 (2)
C1—H1	0.9500	C12—H12	0.9500
C2—C3	1.375 (3)	C13—C14	1.379 (3)
C2—H2	0.9500	C13—H13	0.9500
C3—C4	1.397 (2)	C14—C15	1.388 (3)
C3—H3	0.9500	C14—H14	0.9500
C4—C5	1.382 (2)	C15—C16	1.385 (2)
C4—H4	0.9500	C15—H15	0.9500
C5—C6	1.493 (2)	C16—C17	1.493 (2)
C6—C7	1.493 (2)	C17—C18	1.497 (2)
C7—H7A	0.9800	C18—H18A	0.9800
C7—H7B	0.9800	C18—H18B	0.9800
C7—H7C	0.9800	C18—H18C	0.9800
C8—C9	1.520 (2)	C19—C20	1.516 (2)
C8—H8A	0.9900	C19—H19A	0.9900
C8—H8B	0.9900	C19—H19B	0.9900
C9—H9A	0.9900	C20—H20A	0.9900
C9—H9B	0.9900	C20—H20B	0.9900
C10—H10A	0.9800	C21—H21A	0.9800
C10—H10B	0.9800	C21—H21B	0.9800
C10—H10C	0.9800	C21—H21C	0.9800
C11—H11A	0.9800	C22—H22A	0.9800
C11—H11B	0.9800	C22—H22B	0.9800
C11—H11C	0.9800	C22—H22C	0.9800
			
N2—Mn1—N1	72.15 (5)	N5—Mn2—N4	71.84 (5)
N2—Mn1—N3	74.89 (5)	N5—Mn2—N6	75.47 (5)
N1—Mn1—N3	142.78 (5)	N4—Mn2—N6	144.23 (5)
N2—Mn1—Cl1	136.71 (4)	N5—Mn2—Cl4	106.59 (4)
N1—Mn1—Cl1	95.96 (4)	N4—Mn2—Cl4	103.53 (4)
N3—Mn1—Cl1	96.25 (4)	N6—Mn2—Cl4	99.31 (4)
N2—Mn1—Cl2	100.72 (4)	N5—Mn2—Cl3	142.11 (4)
N1—Mn1—Cl2	102.43 (4)	N4—Mn2—Cl3	98.91 (4)
N3—Mn1—Cl2	100.22 (4)	N6—Mn2—Cl3	97.95 (4)
Cl1—Mn1—Cl2	122.565 (18)	Cl4—Mn2—Cl3	111.302 (17)
C1—N1—C5	118.48 (14)	C12—N4—C16	118.60 (14)
C1—N1—Mn1	125.20 (11)	C12—N4—Mn2	124.80 (11)
C5—N1—Mn1	116.24 (11)	C16—N4—Mn2	116.58 (11)
C6—N2—C8	122.33 (14)	C17—N5—C19	123.01 (14)
C6—N2—Mn1	120.52 (11)	C17—N5—Mn2	120.56 (11)
C8—N2—Mn1	117.11 (10)	C19—N5—Mn2	116.24 (10)
C11—N3—C10	109.47 (15)	C22—N6—C20	110.98 (13)
C11—N3—C9	111.13 (14)	C22—N6—C21	108.69 (13)
C10—N3—C9	109.52 (14)	C20—N6—C21	109.77 (14)
C11—N3—Mn1	112.15 (11)	C22—N6—Mn2	111.41 (10)
C10—N3—Mn1	111.44 (11)	C20—N6—Mn2	104.36 (9)
C9—N3—Mn1	102.98 (10)	C21—N6—Mn2	111.59 (10)
N1—C1—C2	122.84 (16)	N4—C12—C13	122.92 (16)
N1—C1—H1	118.6	N4—C12—H12	118.5
C2—C1—H1	118.6	C13—C12—H12	118.5
C3—C2—C1	118.86 (16)	C14—C13—C12	118.55 (17)
C3—C2—H2	120.6	C14—C13—H13	120.7
C1—C2—H2	120.6	C12—C13—H13	120.7
C2—C3—C4	118.98 (16)	C13—C14—C15	119.07 (16)
C2—C3—H3	120.5	C13—C14—H14	120.5
C4—C3—H3	120.5	C15—C14—H14	120.5
C5—C4—C3	118.98 (15)	C16—C15—C14	119.31 (16)
C5—C4—H4	120.5	C16—C15—H15	120.3
C3—C4—H4	120.5	C14—C15—H15	120.3
N1—C5—C4	121.86 (15)	N4—C16—C15	121.54 (16)
N1—C5—C6	115.07 (14)	N4—C16—C17	115.15 (14)
C4—C5—C6	123.05 (14)	C15—C16—C17	123.29 (15)
N2—C6—C7	125.25 (15)	N5—C17—C16	115.24 (14)
N2—C6—C5	115.62 (14)	N5—C17—C18	125.93 (16)
C7—C6—C5	119.12 (14)	C16—C17—C18	118.83 (15)
C6—C7—H7A	109.5	C17—C18—H18A	109.5
C6—C7—H7B	109.5	C17—C18—H18B	109.5
H7A—C7—H7B	109.5	H18A—C18—H18B	109.5
C6—C7—H7C	109.5	C17—C18—H18C	109.5
H7A—C7—H7C	109.5	H18A—C18—H18C	109.5
H7B—C7—H7C	109.5	H18B—C18—H18C	109.5
N2—C8—C9	107.72 (13)	N5—C19—C20	108.22 (13)
N2—C8—H8A	110.2	N5—C19—H19A	110.1
C9—C8—H8A	110.2	C20—C19—H19A	110.1
N2—C8—H8B	110.2	N5—C19—H19B	110.1
C9—C8—H8B	110.2	C20—C19—H19B	110.1
H8A—C8—H8B	108.5	H19A—C19—H19B	108.4
N3—C9—C8	112.06 (14)	N6—C20—C19	112.10 (14)
N3—C9—H9A	109.2	N6—C20—H20A	109.2
C8—C9—H9A	109.2	C19—C20—H20A	109.2
N3—C9—H9B	109.2	N6—C20—H20B	109.2
C8—C9—H9B	109.2	C19—C20—H20B	109.2
H9A—C9—H9B	107.9	H20A—C20—H20B	107.9
N3—C10—H10A	109.5	N6—C21—H21A	109.5
N3—C10—H10B	109.5	N6—C21—H21B	109.5
H10A—C10—H10B	109.5	H21A—C21—H21B	109.5
N3—C10—H10C	109.5	N6—C21—H21C	109.5
H10A—C10—H10C	109.5	H21A—C21—H21C	109.5
H10B—C10—H10C	109.5	H21B—C21—H21C	109.5
N3—C11—H11A	109.5	N6—C22—H22A	109.5
N3—C11—H11B	109.5	N6—C22—H22B	109.5
H11A—C11—H11B	109.5	H22A—C22—H22B	109.5
N3—C11—H11C	109.5	N6—C22—H22C	109.5
H11A—C11—H11C	109.5	H22A—C22—H22C	109.5
H11B—C11—H11C	109.5	H22B—C22—H22C	109.5

### Hydrogen-bond geometry (Å, °)

**Table:**

*D*—H···*A*	*D*—H	H···*A*	*D*···*A*	*D*—H···*A*
C4—H4···Cl4^i^	0.95	2.73	3.6115 (18)	155
C7—H7B···Cl3^i^	0.98	2.75	3.7280 (18)	175
C14—H14···Cl4^ii^	0.95	2.82	3.7048 (18)	156
C19—H19A···Cl3^iii^	0.99	2.64	3.5839 (18)	159
C19—H19B···Cl4^i^	0.99	2.73	3.6579 (19)	156
C22—H22B···Cl4^i^	0.98	2.78	3.6693 (19)	151

Symmetry codes: (i) −*x*+1, *y*+1/2, −*z*+3/2; (ii) −*x*+1, −*y*+1, −*z*+2; (iii) −*x*+1, *y*−1/2, −*z*+3/2.
